# Hair growth-promoting activity of hot water extract of *Thuja orientalis*

**DOI:** 10.1186/1472-6882-13-9

**Published:** 2013-01-10

**Authors:** Nan-nan Zhang, Dong Ki Park, Hye-Jin Park

**Affiliations:** 1 Department of Bioscience and Biotechnology, Konkuk University, 1 Kwayang-dong, Gwangjin-gu, Seoul, 143-701, Republic of Korea; 2 Cell Activation Research Institute, Konkuk University, 1 Hwayang-dong, Kwangjin-gu, Seoul, 143-701, Republic of Korea

**Keywords:** Anagen, β-catenin, Hair follicles, Hair growth, Sonic hedgehog (Shh), *Thuja orientalis*

## Abstract

**Background:**

*Thuja orientalis* has been traditionally used to treat patients who suffer from baldness and hair loss in East Asia. The present study sought to investigate the hair growth-promoting activity of *T*. *orientalis* hot water extract and the underlying mechanism of action.

**Methods:**

After *T*. *orientalis* extract was topically applied to the shaved dorsal skin of telogenic C57BL/6 N mice, the histomorphometric analysis was employed to study induction of the hair follicle cycle. To determine the effect of *T*. *orientalis* extract on the telogen to anagen transition, the protein expression levels of β-catenin and Sonic hedgehog (Shh) in hair follicles were determined by immunohistochemistry.

**Results:**

We observed that *T*. *orientalis* extract promoted hair growth by inducing the anagen phase in telogenic C57BL/6 N mice. Specifically, the histomorphometric analysis data indicates that topical application of *T*. *orientalis* extract induced an earlier anagen phase and prolonged the mature anagen phase, in contrast to either the control or 1% minoxidil-treated group. We also observed increases in both the number and size of hair follicles of the *T*. *orientalis* extract-treated group. Moreover, the immunohistochemical analysis reveals earlier induction of β-catenin and Shh proteins in hair follicles of the *T*. *orientalis* extract-treated group, compared to the control or 1% minoxidil-treated group.

**Conclusion:**

These results suggest that *T*. *orientalis* extract promotes hair growth by inducing the anagen phase in resting hair follicles and might therefore be a potential hair growth-promoting agent.

## Background

Hair loss is an emotionally distressing disease in humans. It is known that diseases, nutritional deficiency, aging, hormone imbalance, and stress can cause hair loss in both men and women [1,2]. To date, the number of patients suffering from hair loss or alopecia has increased dramatically [3]. Although 2 hair loss drugs, finasteride and minoxidil, have been approved by the Food and Drug Administration, their efficacies are limited and transient, due to unpredictable efficacies and side effects. Therefore, it is urgent to develop more and better treatment options.

Hair, a complex mini-organ composed of terminally differentiated and dead keratinocytes, plays several roles in physical protection, sensory, thermoregulation, and sexual attractiveness. The cyclical process of hair growth is divided into 3 following phases: anagen (growth phase), catagen (regression phase), and telogen (resting phase) [4]. Dysregulation of the hair growth cycle has been shown to be associated with the pathogenesis of certain conditions, for example, androgenetic alopecia. Two key regulators of hair follicle growth, Sonic hedgehog (Shh) and β-catenin, are known to be involved in the induction of the transition from telogen to anagen, and when the level of either protein is low, hair growth is severely damaged [5,6].

*Thuja orientalis* is a distinct genus of evergreen coniferous tree in the cypress family *Cupressaceae* and is distributed widely in China, Japan, and Korea. It has been traditionally used to promote hair growth in the oriental medicine. While *T*. *occidentalis* (Western *T*. *orientalis*) was found to contain a strong 5α-reductase inhibitor that suppresses the peripheral conversion of testosterone into dihydrotestosterone (DHT), it was reported that flavonoid and diterpene from *T*. *orientalis* can be used as 5α-reductase inhibitors for treating androgen-related diseases [7]. 5α-reductase, an enzyme that converts testosterone to DHT, has been suggested to trigger androgenetic alopecia in individuals who are genetically susceptible [1]. A genetically predisposed person, whose follicles are continuously exposed to DHT, has a shorter anagen phase (i.e., hair growth phase) [2]. Increased levels of DHT and 5α-reductasecause the balding scalp skin [2]. To date, the mechanism responsible for the hair promoting effect of *T*. *orientalis* remains unknown. In the present study, we investigated the hair growth-promoting activities of *T*. *orientalis* extract in telogenic C57BL/6 N mice and the underlying mechanism of action.

## Methods

### Materials

The ImmunoCruz Staining System Kit (Santa Cruz Biotechnology, Santa Cruz, California, USA) and the DAB Chromogen Kit (Vector Laboratories, Burlingame, California, USA) were purchased from indicated sources. Antibodies and reagents used in this study were as follows: anti-β-catenin (Cell Signaling Technology, Danvers, MA, USA), anti-Sonic hedgehog (Shh) (Abnova, Taipei, Taiwan), hematoxylin (BBC Biochemical, Mount Vernon, Washington, USA), eosin (Shimakyu’s Pure Chemicals, Osaka, Japan), dimethyl sulfoxide (DMSO) and propylene glycol (Sigma-aldrich, California, USA), and minoxidil (Hyundai Pharm, Seoul, South Korea).

### Preparation of *T*. *orientalis* hot water extract

An authenticated voucher specimen of *T*. *orientalis* leaves (Kucari 1108) was deposited in the Herbarium of the College of Bioscience and Biotechnology, Konkuk University (Seoul, Korea). Leaves were ground to a fine powder with a grinder and extracted 4 times with hot water for 4 h. Hot water extract was then chilled, filtered through the Advantech No. 2 filter paper (Osaka, Japan), and allowed to evaporate to dryness. Residues were extracted with hot water again at room temperature and filtered. Extract was dried in a rotary evaporator under vacuum at 40°C and subsequently stored at −20°C until use. *T*. *orientalis* extract was dissolved in water for animal experiments [8].

### Experimental animals

Male C57BL/6 N mice (5-week-old, 18–20 g) purchased from Orient Bio Inc. (Seoul, South Korea) were cared in a controlled barrier facility within the Konkuk University Laboratory Animal Research Center. Mice were housed in cages under a condition of 12-h light/dark cycle and maintained on standard mouse chow and water. The room temperature and humidity were 23 ± 2°C and 35–60%, respectively. After mice were adapted to their new environment for 7 days, experiments were carried out using 6-week-old mice, since 6- to 9-week-old C57BL/6 N mice were shown to be in the telogen stage of hair cycle [9,10]. All animal procedures were performed according to the Guide for the Care and Use of Laboratory Animals of the National Institutes of Health, as well as the guidelines of the Animal Welfare Act. All experiments were carried out in accordance with the guidelines of The Institutional Animal Care and Use Committee (IACUC) at Konkuk University (Seoul, Korea). The protocol ku11069 was approved by Konkuk University Medical center IACUC for this study.

### Experimental studies with *T*. *orientalis* extract

Thirty animals in 3 randomized groups (*n* = 10) were used for studying the hair promoting activity of *T*. *orientlis* extract. A 12-cm^2^ area (horizontal length, 3 cm; longitudinal length, 4 cm) of hair was shaved from the dorsal portion of C57BL/6 N mice with an animal clipper at 6 weeks of age, at which mouse hair follicles were synchronized in the telogen stage. While animals in group 1 received distilled water (200 μl) with an equal volume of mixture containing propylene glycol (96.5%, v/v) and DMSO (3.5%, v/v), animals in groups 2 and 3 received *T*. *orientalis* extract and 1% minoxidil (200 μl), respectively, with an equal volume of the same mixture described. *T*. *orientalis* extract or vehicle was applied topically on the dorsal skin for 21 days using a syringe plunger with the same strokes. Animals were kept in isolation for a certain amount of time and then housed back to separate cages. At 0, 7, 14, and 21 days, mice were sacrificed to obtain skin specimens. Visible hair growth was recorded at 0, 7, 10, 14, 17, and 21 days.

### Hair length determination

Regrown hairs were plucked from representative areas in shaved dorsal center parts of sacrificed mice on 14 and 21 days. We calculated the average hair length from 30 hairs per mouse.

### Histological preparation

Dorsal skin of mice was fixed with 10% neutral buffered formalin at 4°C for 24 h and washed with PBS (pH 7.4). Fixed samples were dehydrated through an ascending series of graded ethanol, cleared in xylene, and embedded in paraffin blocks. Subsequently, samples were cut either longitudinally or transversely into 5-μm-thick sections and mounted on gelatin-coated glass slides.

### Quantitative histomorphometry

Skin biopsies were fixed with 10% neutral formalin for routine histology, paraffin-embedded, and processed for hematoxylin-eosin staining. Individual hair follicles were confined to specific hair cycle stages (telogen or anagen I–VI), based on the classification of Chase [11]. The percentage of hair follicles in each defined cycle stage at 7, 14, and 21 days was calculated.

### Hematoxylin-eosin staining

To observe the histological change after topical application of *T*. *orientalis* extract, sections were stained with hematoxylin and eosin. Briefly, sections were deparaffinized with xylene, hydrated in a descending series of graded ethanol, and stained with hematoxylin for 2 min, followed by washes for 2 min and eosin staining for 5 s.

### Hair follicle counting

Digital photomicrographs were taken from representative areas of slides at a fixed magnification of 100 ×. All images were cropped in a fixed area with a width of 1500 um. We then manually counted hair follicles in deep subcutis (*n* = 30/mouse).

### Immunohistochemistry

Dorsal skins were stained with anti-β-catenin and anti-Shh antibodies, as previously described [12]. The immunohistochemical analysis was performed using the ImmunoCruz Staining System Kit and DAB Chromogen Kit, according to the manufacturer’s instructions.

### Statistical analysis

The experimental data were expressed as mean ± standard deviation (S.D.). The significance of differences was analyzed using the Student’s *t*-test or One-way ANOVA/Dunnett’s *t*-test. We used SPSS, version 12 (SPSS Inc., Chicago, IL, USA) for the statistical analysis.

## Results

### Hot water extract of *T*. *orientalis* promotes hair growth in telogenic C57BL/6 N mice

**Figure 1:**
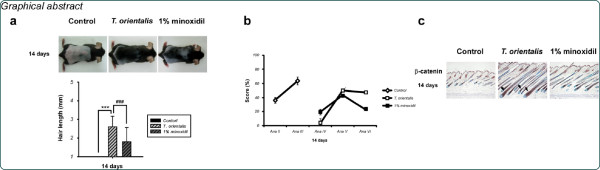
**Hair growth-promoting effects of *Thuja orientalis *extract.** (**a**) Telogen-matched, 6-week-old C57BL/6 N mice were shaved and topically applied with control (*n* = 10; A–F), *T*. *orientalis* extract (5.05 mg/cm^2^/day; *n* = 10; G–L), or 1% minoxidil (*n* = 10; M–R). The back skins were photographed at 0, 7, 10, 14, 17, and 21 days after depilation. The image shown is a representative picture of 10 mice. Bars, 100 μm. (**b**) Hair lengths at different time intervals after treatment with *T*. *orientalis* extract. Data shown represent means ± standard deviation (S.D.) of 3 independent experiments. Comparisons of multiple group means were performed using One-way ANOVA, followed by the Dunnett’s *t*-test (^***^p < 0.001, vs. control; ^###^*p* < 0.001, vs. 1% minoxidil).

To measure the hair growth activity of *T*. *orientalis* extract *in vivo*, telogenic C57BL/6 N mice were shaved 1 day before topical application of *T*. *orientalis* extract. The skin color of mice in the telogen phase was pink and became dark along with anagen initiation [13]. Since the active growth of hair follicles and black pigmentation occur in C57BL/6 N mice during the anagen phase [14], the hair growth-promoting activity of *T*. *orientalis* extract was evaluated by observing the skin color. More blacken skin areas were observed in *T*. *orientalis* extract-treated group at 10 days, compared to the control or 1% minoxidil group (Figure 1a). At 14 days, we observed that *T*. *orientalis* extract promoted hair growth more prominently than either the control or 1% minoxidil group (Figure 1a). At 17 days, dorsal skin hairs were fully recovered in *T*. *orientalis* extract-treated mice (Figure 1a), whereas only 50% of the dorsal skin area in the control group was covered with hairs (Figure 1a). These results suggest that *T*. *orientalis* extract induces early telogen-to-anagen conversion of hair follicles.

To determine whether *T*. *orientalis* extract induces hair growth, we plucked 30 hairs from the dorsal skin center area of each mouse at both 14 and 21 days. Our results show that *T*. *orientalis* extract significantly stimulated hair growth, compared to the control group, and that the hair length of *T*. *orientalis* extract-treated mice was significantly longer than that of the control or 1% minoxidil-treated group at 14 days (p < 0.001) (Figure 1b).

### Effects of *T*. *orientalis* extract on the development and structure of mouse hair follicles

**Figure 2:**
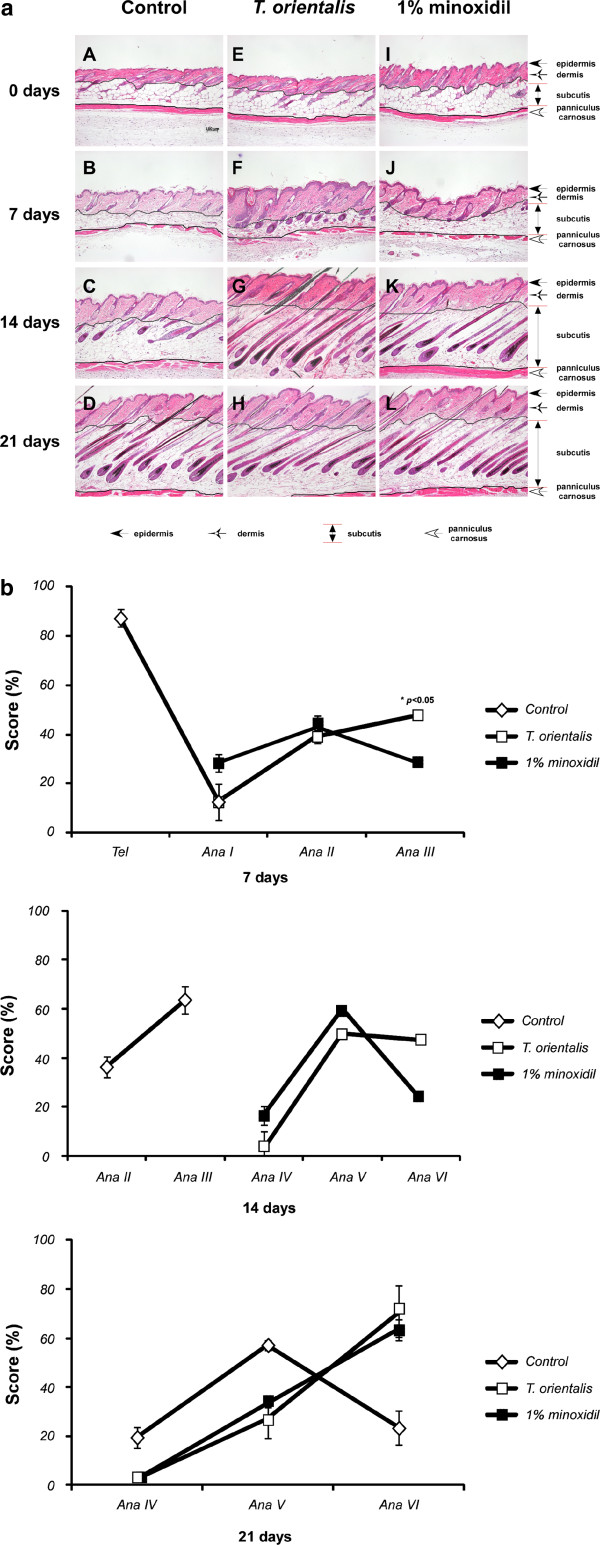
**Hair follicle growth in telogen-matched C57BL/6 N mice treated with *T. orientalis *extract.** The effect of *T*. *orientalis* extract (5.05 mg/cm^2^/day) on the hair follicles in telogen mice was analyzed using hematoxylin-eosin (H&E) staining. (**a**) Longitudinal sections of the back skins (0, 7, 14, and 21 days) were stained, and the image shown is a representative picture of 10 mice. Bars, 100 μm. A–D, control; E–H, *T*. *orientalis* extract; I–L, 1% minoxidil. (**b**) Quantitative histomorphometric analysis. *Y* axis, hair cycle score value; *X* axis, progress of hair follicles in the hair cycle, assessed by histomorphometry: telo, telogen; ana I–VI, anagen I to VI; *left*, hair cycle score values. Values are mean ± standard deviation (S.D.) (*n* = 30 hair follicles/mouse; ^*^p < 0.05, vs. 1% minoxidil).

An increase in the number and size of hair follicles has been considered as an indicator for the transition of hair growth from the telogen to anagen phases [15]. To investigate the progression of hair follicles in the hair cycle, hematoxylin-eosin staining was performed, since an increase in the size and number of hair follicles can be observed in the deep subcutis during the anagen phase [15]. In the representative longitudinal sections, the number of hair follicles was increased in *T*. *orientalis* extract-treated group, compared to the control group (Figure 2a). To quantify the hair promoting effects, we performed the histomorphometric analysis. Individual hair follicles were classified following the Chase’s protocol [16]. At day 7, the majority of hair follicles in *T*. *orientalis* extract-treated group progressed to the anagen stages II–III, whereas the majority in control group remained in the telogen stage (Figure 2b). At day 14, while the hair follicles of *T*. *orientalis* extract-treated group were in anagen V–VI, those of minoxidil treated- and control groups were in anagen V and III, respectively. At day 21, the hair follicles in both *T*. *orientalis* extract- and 1% minoxidil-treated groups were in anagen VI, whereas the control group remained in anagen V. These results sugest that topical application of *T*. *orientalis* extract could induce an earlier anagen phase and prolong the mature anagen phase, compared to either the control or 1% minoxidil-treated group (Figure 2b).

**Figure 3:**
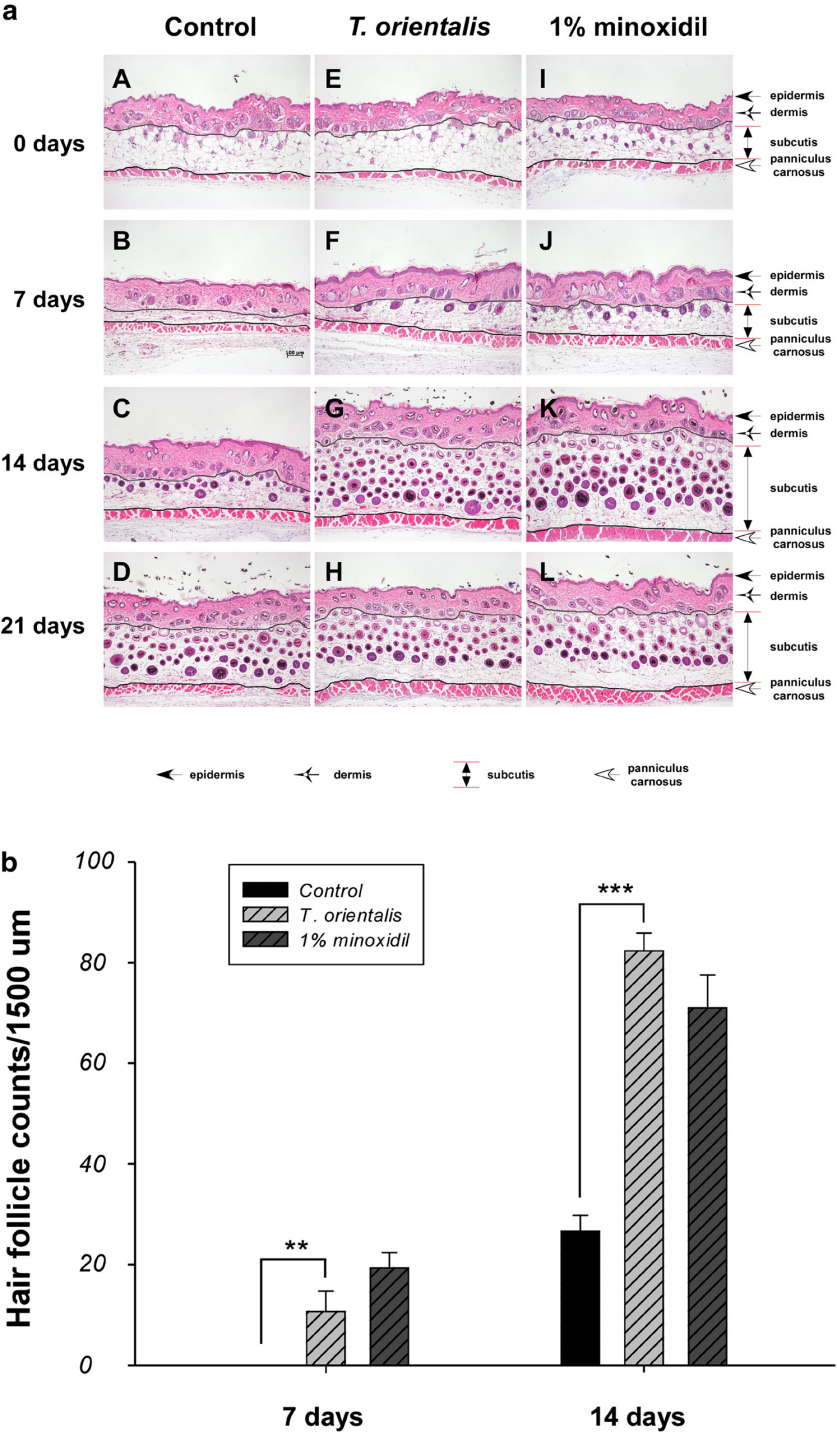
**Hair follicle growth in telogen**-**matched C57BL**/**6 N mice treated with *T. orientalis *extract.** The effect of *T*. *orientalis* extract (5.05 mg/cm^2^/day) on the hair follicles of telogen mice was analyzed using hematoxylin-eosin (H&E) staining. (**a**) Transverse sections of the back skins (0, 7, 14, and 21 days) were stained, and representative photomicrographs of skin sections were shown. Bars, 100 μm. A–D, control; E–H, *T*. *orientalis* extract; I–L, 1% minoxidil. (**b**) The number of hair follicles in deep subcutis. Values are mean ± standard deviation (S.D.) (*n* = 10/mouse; ^**^p < 0.01 and ^***^P < 0.001, vs. control).

In addition, topical application of *T*. *orientalis* extract also significantly increased the number of hair follicles in mice, compared to the control group at 7 and 14 days (Figure 3a). At 7 and 14 days, the number of hair follicles in deep dermal areas of *T*. *orientalis* extract-treated group was greater than that in the control group (p < 0.001) (Figure 3b).

### Induction of the anagen phase by *T*. *orientalis* extract in telogenic C57BL/6 mice

**Figure 4:**
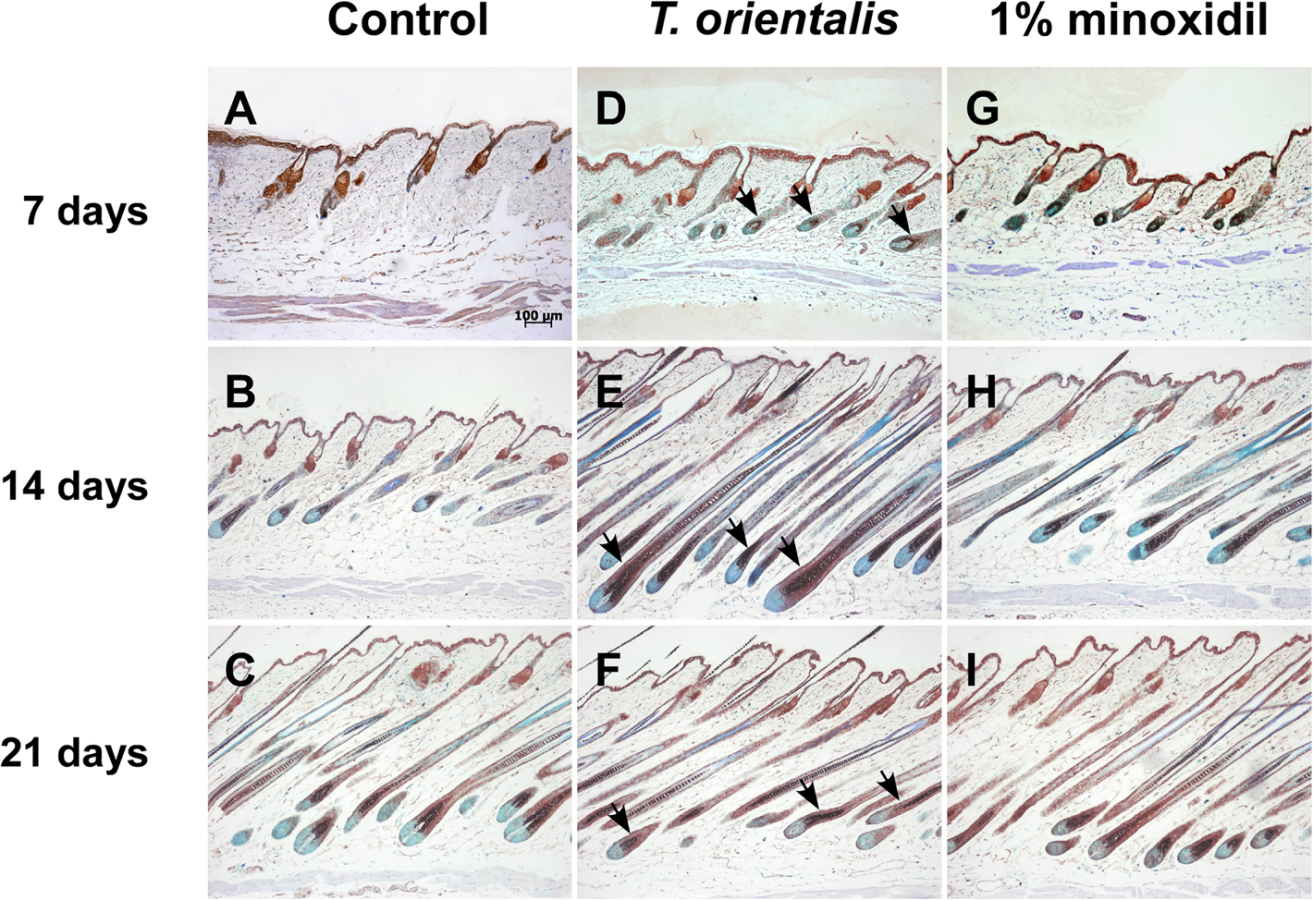
**Induction of β**-**catenin protein expression after topical application of *T. orientalis *extract**. The immunohistochemical analysis was used to monitor the protein expression of β-catenin (arrows) in the longitudinal sections of dorsal skins (7, 14, and 21 days). Bars, 100 μm. **A**–**C**, control; **D**–**F**, *T*. *orientalis* extract; **G**–**I**, 1% minoxidil.

**Figure 5:**
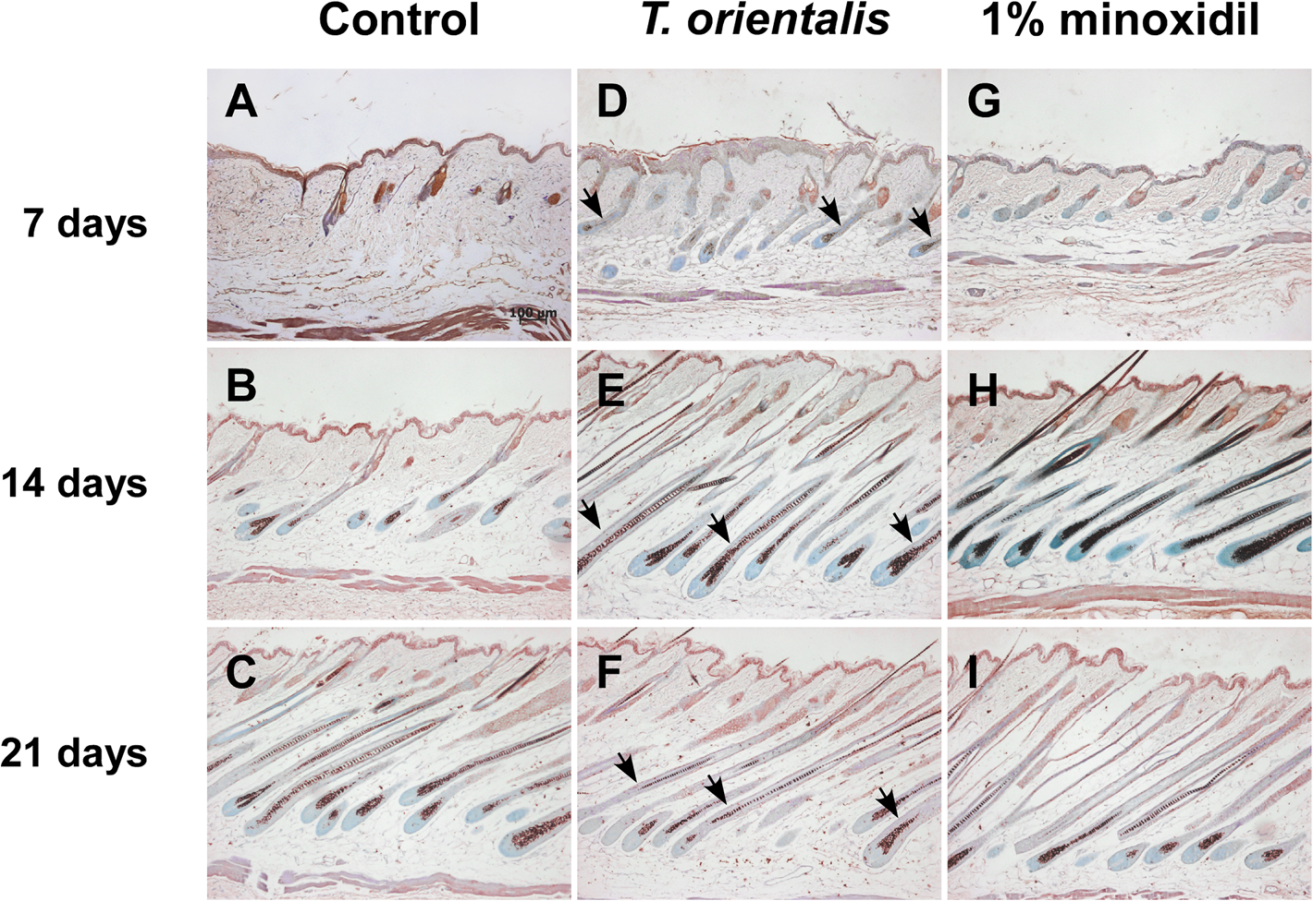
**Induction of Sonic hedgehog** (**Shh**) **protein expression after topical application of *T. orientalis *extract**. The immunohistochemical analysis was used to monitor the protein expression of Sonic hedgehog (Shh) (arrows) in the longitudinal sections of dorsal skins (7, 14, and 21 days). Bars, 100 μm. **A**–**C**, control; **D**–**F**, *T*. *orientalis* extract; **G**–**I**, 1% minoxidil.

To elucidate the mechanism underlying the induction of anagen phases in *T*. *orientalis* extract-treated group, we performed the immunohistochemistry analysis using anti-β-catenin and anti-sonic hedgehog (Shh) antibodies. Previously, it has been reported that both β-catenin and Shh proteins are essential for the development and maintenance of hairs not only in embryos, but also in adults [17]. Several studies also showed that β-catenin and Shh induced the transition of the hair growth cycle from the telogen to anagen phases [11] and that transient activation of β-catenin induced the anagen phase [18]. Here, we demonstrate that the protein level of β-catenin in *T*. *orientalis* extract-treated group at 14 days was higher than that in the control or minoxidil-treated group (Figure 4). Moreover, Shh is known to be expressed in inner root sheath and outer root sheath, sebaceous gland, hair follicles, and epidermis [6,15]. We observed that the protein level of Shh at 14 days was also higher in *T*. *orientalis* extract-treated group, compared to the control group (Figure 5).

### Chromatogram of T.orientalis extract

**Figure 6:**
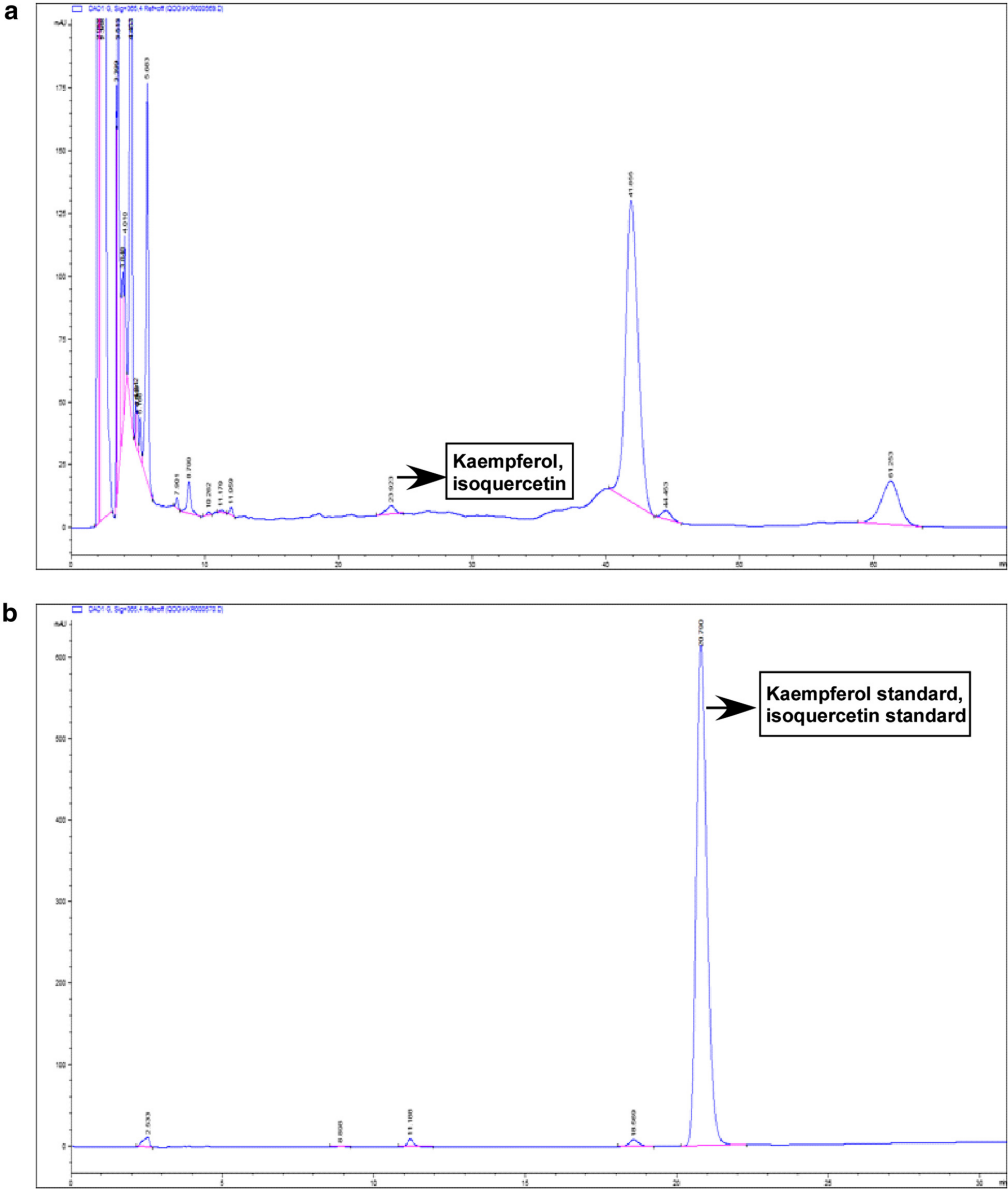
**High performance liquid chromatography** (**HPLC**) **chromatogram of a**) **a hot water extract of *Thuja orientalis *and b**) **kaempferol and isoquercetin standard.**

HPLC chromatogram indicated that kaempferol and isoquercetin were found in hot water extract of *Thuja orientalis* leaves. It has been reported that kaempferol or isoquercetin, a polyphenolic flavonoid, possesses antioxidants [19-21], anti-inflammatory [22] and inhibitory activity in cellular events, which associated with initiation, promotion and progression of carcinogenesis [23,24]. These activities of two components might be contributed to hair promoting activity of *Thuja orientalis* extract (Figure 6).

## Discussion

Hair loss disorders, although are not life-threatening, are emotionally distressing diseases that make afflicted patients vulnerable. While minoxidil has been reported to be efficacious in promoting hair growth in androgenic alopecia patients by inducing hair follicles in the telogen stage to undergo transition into the anagen stages [25], the drug would also cause adverse dermatological effects, such as pruritis, dryness, scaling, local irritation, and dermatitis [26]. Due to the undesirable side-effects and low efficacy for treating hair loss or hair thinning, the therapeutic uses of conventional drugs have been limited. On the other hand, increased attention has been being paid to herbal medicines that could exert their hair promoting activity, with minimal or no side effects or toxicities. Several traditional herbal medicines have been widely used for treating diseases or preventing hair loss in Far East Asia. For instance, *T*. *orientalis* Linn has been used to treat gout, rheumatism, diarrhea, and chronic tracheitis [27-29]. Recently, *T*. *orientalis* was shown to not only act as 5α-reductase inhibitors for treating androgen-related diseases but also possess biological activities, including antioxidant and anti-elastase activities [30], as well as anti-inflammatory functions [29]. However, no study has looked at the mechanism of the hair growth-promoting activity of *T*. *orientalis* hot water extract. In this current study, we investigated the hair growth-promoting activity of *T*. *orientalis* extract using 6-week-old C57BL/6 N mice in the stable telogen phase. C57BL/6 N mice are useful for screening hair growth-promoting agents, because their truncal pigmentation is dependent on their follicular melanocytes, which produce pigment only during anagen [31]. The shaved back skins of C57BL/6 N were topically applied with *T*. *orientalis* extract for 7, 10, 14, 17, and 21 days. At 14 days, *T*. *orientalis* extract significantly induced hair growth in telogenic C57BL/6 N mice, whereas little visible hair growth was observed in the control group (Figure 1a). To further investigate the hair growth-promoting effect, we randomly plucked 30 hairs from the center area of each mouse and measured the hair length. We found that the hair length of *T*. *orientalis* extract-treated group was significantly longer than that of the control group (Figure 1b). Moreover, the histomorphometric analysis data indicate that topical application of *T*. *orientalis* extract caused an earlier induction of the anagen phase, compared to either the control or 1% minoxidil-treated group (Figure 2a).

It is known that various hormones, growth factors, and development-related molecules are involved in hair growth [13]. In addition, elevated levels of several activators have also been observed in hair follicles that were in the anagen phase [32]. Among these activators, β-catenin and Sonic hedgehog (Shh) are key regulators of hair follicle growth and cycling. Both proteins have been reported to induce the transition of hair follicles from the telogen to anagen phase [14,33], and the level of Shh protein was also found to be significantly decreased when hair follicles entered the catagen phase [6]. To elucidate the molecular mechanism underlying the ability of *T*. *orientalis* extract to induce anagen hair follicles, we examined the protein levels of β-catenin and Shh in the shaved dorsal skin at 7, 14, and 21 days. Our immunohistochemical analysis results show that the expression levels of β-catenin and Shh were upregulated in *T*. *orientalis* extract-treated group at 14 days, compared to those in the control or 1% minoxidil-treated group (Figures 4 and 5). Interestingly, some studies have previously suggested that continuous β-catenin signaling may cause hair follicle tumors [17]. At 21 days, however, we observed that protein levels of β-catenin and Shh were gradually decreased in *T*. *orientalis* extract and minoxidil-treated groups (Figures 4 and 5), indicating that *T*. *orientalis* extract did not continuously induce the anagen phase of hair follicles. HPLC chromatogram showed that kaempferol and isoquercetin were contained in *Thuja orientalis* extract. However, we cannot rule out the possibility that other components in a hot water extract of *Thuja orientalis* exert hair promoting activity. Further chemical screening analysis for the other bioactive components in *Thuja orientalis* extract will help to understand the detailed mechanism of its hair promoting activity.

Further detailed clinical trials and studies will be necessary to investigate what components in *T*. *orientalis* extract contribute to its efficacy, since whole *T*. *orientalis* extract, rather than individual components, was used here to prove its biological activity against pathogenic alopecia.

## Conclusion

In conclusion, our report is the first to show that hot water extract of *T*. *orientalis* promoted hair growth by inducing anagen in telogenic C57BL/6 N mice. In *T*. *orientalis* extract-treated mice, we observed an increase in the number and size of hair follicles, which served as a piece of evidence for the induction of anagen phases. Using the immunohistochemical analysis, we observed an earlier induction of β-catenin and Shh proteins in *T*. *orientalis* extract-treated group, compared to the control or 1% minoxidil-treated group. Taken together, these results suggest that *T*. *orientalis* extract promotes hair growth by inducing the anagen phase of hair follicles and might therefore be a potential hair promoting agent.

## Competing interests

The authors have declared that there is no conflict of interests.

## Authors’ contributions

NZ carried out the molecular analysis and immunohistochemistry. DKP participated in the design of the study and performed the statistical analysis. HJP carried out the molecular analysis, as well as conceived the study, participated in its design and coordination, and helped to draft the manuscript. All authors read and approved the final manuscript.

## Pre-publication history

The pre-publication history for this paper can be accessed here:

http://www.biomedcentral.com/1472-6882/13/9/prepub
